# Surface-treated self-standing curved crystals as high-efficiency elements for X- and γ-ray optics: theory and experiment^1^

**DOI:** 10.1107/S1600576715006809

**Published:** 2015-04-25

**Authors:** Elisa Bonnini, Elisa Buffagni, Andrea Zappettini, Stephen Doyle, Claudio Ferrari

**Affiliations:** a IMEM Institute, National Research Council, Parco Area delle Science 37/A, Parma 43124, Italy; b Department of Physics and Earth Sciences, University of Parma, Parco Area delle Science 7/A, Parma 43124, Italy; c MIST E-R Laboratory S.c.r.l., via P. Gobetti 101, Bologna 40129, Italy; d Synchrotron Light Source ANKA, Hermann-von-Helmholtz-Platz 1, Eggenstein-Leopoldshafen 76344, Germany

**Keywords:** X-ray focusing lenses, integrated reflectivity, curved crystals, crystal stacks

## Abstract

The efficiency of a Laue lens for X- and γ-ray focusing in the energy range 60–600 keV is closely linked to the diffraction efficiency of the single crystals composing the lens. A powerful focusing system is crucial for applications like medical imaging and X-ray astronomy where wide beams must be focused. Mosaic crystals with a high density, such as Cu or Au, and bent crystals with curved diffracting planes (CDPs) are considered for the realization of a focusing system for γ-rays, owing to their high diffraction efficiency in a predetermined angular range. In this work, a comparison of the efficiency of CDP crystals and Cu and Au mosaic crystals was performed on the basis of the theory of X-ray diffraction. Si, GaAs and Ge CDP crystals with optimized thicknesses and moderate radii of curvature of several tens of metres demonstrate comparable or superior performance with respect to the higher atomic number mosaic crystals generally used. In order to increase the efficiency of the lens further, a stack of several CDP crystals is proposed as an optical element. CDP crystals were obtained by a surface-damage method, and a stack of two surface-damaged bent Si crystals was prepared and tested. Rocking curves of the stack were performed with synchrotron radiation at 19 keV to check the lattice alignment: they exhibited only one diffraction peak.

## Abstract 2

It is demonstrated that crystals with curved diffracting planes made of relatively light elements, such as silicon, germanium and gallium arsenide, in the Laue diffraction geometry and in an energy range between 60 and 600 keV can reach a higher diffraction efficiency than more dense mosaic crystals such as copper, silver and gold. In particular, self-standing curved crystals can be used as elements in γ-ray lenses.

## 1. Introduction   

The possibility of focusing γ-rays in the energy range between 60 and 600 keV is extremely important in astronomy to improve the sensitivity of telescopes (Frontera *et al.*, 2005 ▶), and in nuclear medicine for both *in vivo* diagnosis of diseases associated with organ malfunction and cancer detection (Roa *et al.*, 2005 ▶). A clear example is given by the case of scintigraphic monitoring, in which target images are currently obtained by collecting monochromatic γ-radiation coming from specific radiopharmaceuticals, selected in the emission energy range from 80 keV (xenon-133) to 511 keV (fluorine-18), by means of γ cameras based on fine multi-hole collimators and scintillators. Unfortunately, in such systems most of the radiation is lost because of collimator absorption.

Bragg diffraction in the Laue geometry is a powerful tool to focus X- and γ-rays at high energy. This is the goal of a Laue lens, which is composed of a set of crystals disposed in concentric rings and properly oriented in order to concentrate the diffracted beams into the focal point on the detector.

Crystals suitable for such a lens should be able to diffract the radiation efficiently over an angular range, depending on the lens resolution, from a few tens of arcseconds up to a few arcminutes. This is the case in scintigraphy, where focusing distances of a few metres are required in order to insert the focusing lens in the currently available scintigraphic machines. Perfect crystals cannot be used, owing to their very narrow angular range of diffraction at γ-ray energies. On the other hand, mosaic crystals are good candidates as optical elements for these lenses, because their angular acceptance can be directly tuned by modifying the mosaic spread and/or the size of the microcrystals forming the mosaic structure during the growth process. Moreover, the integrated reflectivity *I*_int_/*I*_0_ (defined as the area of the diffraction profile for monochromatic radiation normalized for the incident beam intensity *I*_0_, giving an indication of the diffraction efficiency of the crystal) of an ideal mosaic crystal can be two orders of magnitude larger than that of a perfect crystal, as predicted by the dynamical theory of X-ray diffraction (Authier & Malgrange, 1998 ▶). Unfortunately, the production of crystals with a well defined mosaic spread and grain size is a difficult technological task, so that the resulting diffracted intensity is often much lower than that predicted for the ideal case. Mosaic crystals of high diffraction efficiency at γ-ray energies, such as Cu, Au, Ag and many others, have been proposed (Lund, 1992 ▶; Courtois *et al.*, 2005 ▶; Barrière *et al.*, 2009 ▶) owing to their high electronic density and structure factors.

An effective alternative is represented by self-standing bent crystals, in which the external curvature also induces a bending of the diffracting planes. In this case, the Bragg condition is satisfied for a broad angular range according to the degree of curvature; this also causes the high angular acceptance of these crystals (Smither *et al.*, 2005 ▶). If the radius of curvature *R* is smaller than a critical value resulting from the dynamical theory of diffraction, curved diffracting plane (CDP) crystals can theoretically reach the values of integrated reflectivity obtained for ideal mosaic crystals (Malgrange, 2002 ▶).

A simple method to obtain self-standing crystals with a reproducible and uniform curvature is based on controlled surface damage induced by a mechanical lapping process. A compressive strained layer a few micrometres thick on the damaged side causes the convex curvature of the crystal (Buffagni *et al.*, 2012 ▶, 2013 ▶). This technique allows one to obtain an external radius of curvature *R* down to 1 m in crystals up to 2 mm thick in the direction perpendicular to the damaged surface. The diffraction profile of the CDPs, parallel to the damaged surface, is close to that of an ideal bent crystal curved by elastic deformation (Ferrari *et al.*, 2014 ▶). Currently, this approach is applied only to semiconducting crystals such as Si, Ge and GaAs, thanks to their mechanical properties and lattice perfection. Up to now, the realization of CDP crystals thicker than a few millimetres has been an open issue. Thick bent GeSi alloy crystals have been obtained by composition grading during Czochralski growth (Erko *et al.*, 1996 ▶), but this method showed a low yield, which makes the technique unsuitable for Laue lenses, where a large number of crystals are required.

In this paper, a theoretical study of the integrated reflectivity of CDP crystals is presented based on the dynamical theory of diffraction. We have aimed to select the best material and the best values for parameters such as density, curvature, dimensions and diffraction geometry, to maximize the diffraction efficiency in the energy range of interest.

A possible solution to obtain thicker crystal elements is also investigated: the stacking of CDP crystals aligned with each other to create one multi-lamellar CDP element. We show experimentally how it is possible to achieve a good alignment of single crystals in a stack of surface-damaged Si(100) crystals.

## 2. Diffraction efficiency of CDP and mosaic crystals   

**Figure 1:**
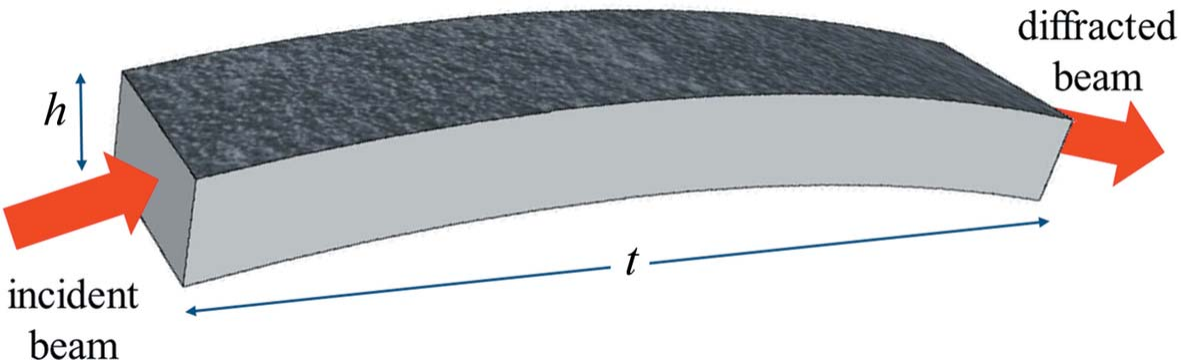
A schematic diagram of a bent crystal in the Laue diffraction geometry. The damaged surface is the upper one, parallel to the CDPs and perpendicular to *R*.

The method of surface damage can produce crystal tiles of a few millimetres in height (indicated as *h* in Fig. 1 ▶), although the length *t* of the tile may be several centimetres. Uniform spherical or elliptical curvatures, depending on the features of the material (Buffagni *et al.*, 2012 ▶), can be obtained along the crystal surface. For example, 30 × 10 × 2 mm GaAs bent tiles were realized by this technique, with a radius of curvature *R* = 40 m along the 30 mm-long direction for a prototype of a Laue lens for astronomical application (Virgilli *et al.*, 2013 ▶; Liccardo *et al.*, 2014 ▶). The present study of diffraction efficiency in bent crystals is performed by considering CDPs parallel to the surface with the incident beam traversing the length *t* of the crystal (see Fig. 1 ▶).

Considering CDP crystals with a uniform curvature, a deformation parameter β can be introduced (Malgrange, 2002 ▶):
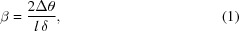
where δ is the Darwin width of the diffraction, defined as the full width at half-maximum (FWHM) of the rocking curve for a perfect crystal, and *l* is the optical path of the beam along the crystal thickness *t*, related by *l* = *t*/(cosθ_B_). Since at high energy the Bragg angle θ_B_ is very small, it is possible to assume *l* = *t*. The variation Δθ of the Bragg condition θ_B_ along the direction of the incident beam along *t* provides an evaluation of the curvature of the diffracting planes, and it is related to the external radius of curvature of the crystal *R* by *R* = *t*/Δθ. Similar but more complicated relationships can be obtained for lattice planes inclined with respect to the crystal surface, taking into account the elastic constants, the variation in lattice spacing along the crystal thickness and the orientation of the crystal (Kalman & Weissmann, 1979 ▶; Ferrari *et al.*, 2013 ▶).

**Figure 2:**
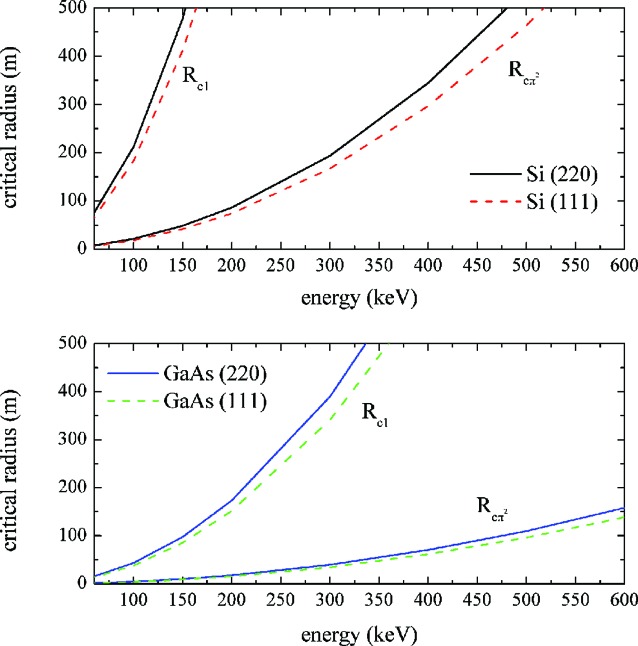
The critical radius of curvature, calculated as a function of energy for the conditions βΛ = 1 (*R*_c1_) and βΛ = π^2^ (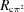) for Si and GaAs (top and bottom panels, respectively), in the case of the 111 and 220 reflections.

Consider the extinction length 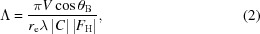
where *V* is the volume of the unit cell of the crystal, *r*_e_ is the classical electron radius, λ is the wavelength of the incident beam, *C* is the polarization factor, *F*_H_ is the structure factor corresponding to the diffracting plane (*hkl*), and the condition βΛ = 1 defines the critical radius of curvature *R*_c1_. When *R* << *R*_c1_, the crystals can be considered strongly bent (βΛ >> 1; Malgrange, 2002 ▶). On the basis of the dynamical theory of diffraction, the radius *R*_c1_ has been calculated as a function of energy over the range of interest in CDP Si and GaAs crystals (Fig. 2 ▶). This shows that the condition of strong curvature occurs for radii of several tens of metres, which are easy to obtain by the lapping process. In this case, the diffraction profile assumes a square shape, with the FWHM given directly by the bending Δθ of the diffracting planes (Malgrange, 2002 ▶), and the peak intensity is
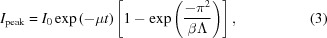
where *I*_0_ is the intensity of the incident beam and μ is the absorption factor of the material.

It is important to note that the peak reflectivity *I*_peak_/[*I*_0_exp(−μ*t*)] may be close to 1, whereas for a mosaic crystal this value cannot exceed 0.5 (Barrière *et al.*, 2009 ▶): this is an intrinsic limitation on the use of mosaic crystals as optical elements in a Laue lens.

The integrated reflectivity *I*_int_/*I*_0_, *i.e.* the area under the reflectivity curve, is considered here, rather than the mere peak reflectivity, as the most relevant parameter characterizing the diffraction efficiency of the crystal. In fact, the peak intensity alone does not take into account the width of the rocking curve, which is strongly influenced by crystal features (mosaicity or curvature).

For strongly bent crystals, owing to the square shape of the rocking curve, the integrated intensity *I*_int_ can be given approximately by *I*_peak_Δθ and, taking into account equation (3), can be written as
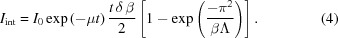
The condition βΛ = π^2^ gives a new critical value *R*_cπ^2^_ (see Fig. 2 ▶). For radii of curvature *R* << *R*_cπ^2^_ (βΛ >> π^2^) the *I*_int_ in equation (4) can be approximated with
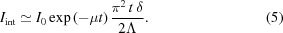

*I*_int_ corresponds to the maximum integrated intensity for CDP crystals. It no longer depends on the crystal curvature and coincides with the *I*_int_ of an ideal mosaic crystal.

**Figure 3:**
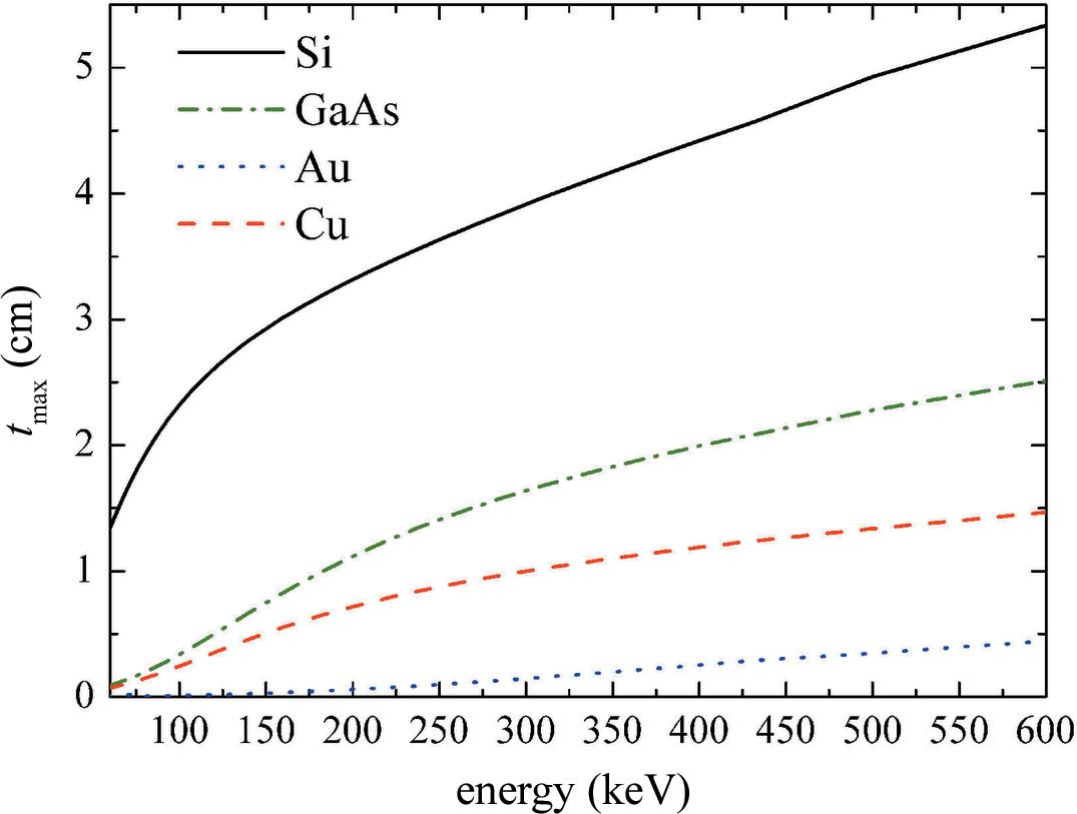
The crystal thickness *t*_max_, maximizing the integrated reflectivity for CDP crystals (Si and GaAs) and mosaic crystals (Cu and Au) in the energy range 60–600 keV.

The crystal thickness *t* traversed by the beam is therefore a crucial parameter for the optimization of integrated reflectivity in mosaic and CDP crystals. The thickness which maximizes the diffraction efficiency, obtained from the partial derivative of *I*_int_ [equation (4)] with respect to *t*, is *t*_max_ = 1/μ, as in the case of symmetrical Laue diffraction for mosaic crystals (Authier & Malgrange, 1998 ▶). Fig. 3 ▶ compares *t*_max_ for ideal mosaic (Cu and Au) and CDP (Si and GaAs) crystals in the energy range of interest; here and below the calculation for Ge is not reported, because of its superposition on that of GaAs. It is important to note that *t*_max_ is almost independent of the reflection geometry and that a high absorption coefficient implies a low crystal thickness for efficiency optimization.

## 3. Diffraction efficiency in Si, Ge and GaAs crystals at soft γ-ray energies   

Considering the expression of the Darwin width *δ*,
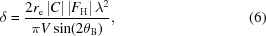
and replacing *t* with *t*_max_ = 1/μ in equation (5), we obtain a formula that expresses the maximum integrated intensity with respect to thickness for ideal mosaic and CDP crystals: 
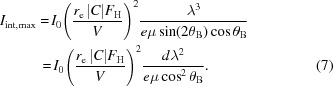
Here, *I*_int,max_ depends on the square of the ratio *F*_H_/*V*, which is proportional to the atomic number *Z* of the crystal. This is the main reason for choosing high-*Z* mosaic crystals such as Cu, Au and Ag (Halloin & Bastie, 2005 ▶).

**Figure 4:**
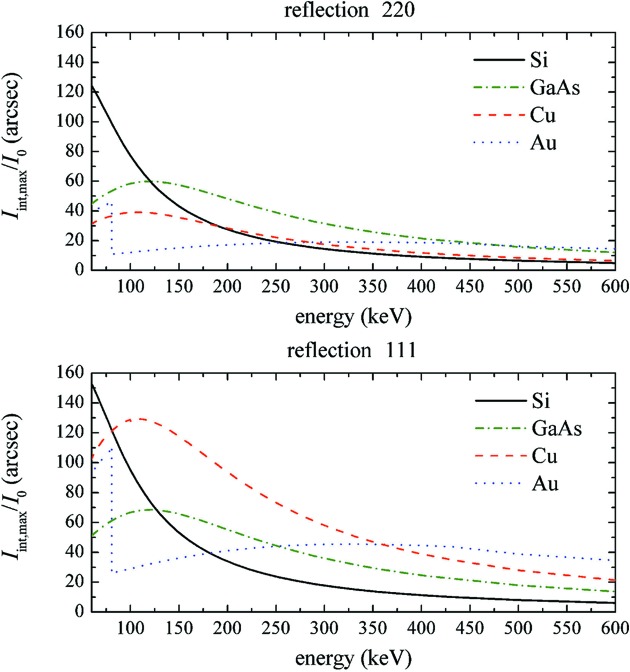
The calculated integrated reflectivity as a function of energy for Si and GaAs CDPs and for Cu and Au mosaic crystals in the 220 and 111 reflections (top and bottom panels, respectively). The thickness of the crystals was tuned to obtain the maximum integrated reflectivity at each energy.

Fig. 4 ▶ shows the maximized integrated reflectivity *I*_int,max_/*I*_0_ calculated from equation (7) for GaAs, Si, and high-density materials such as Cu and Au usually proposed as γ-ray lens elements; the diffraction geometries with the highest values of *F*_H_ for face-centred cubic crystals are considered, *i.e.* the 111 and 220 reflections. Data concerning the absorption coefficients of the materials considered have been taken from the NIST database (http://www.nist.gov/pml/data/index.cfm).

Equation (7) is valid only for strongly bent crystals with βΛ >> π^2^, so that lower curvatures (larger *R*) give lower values of the integrated intensity. For instance, for βΛ = π^2^/2 and βΛ = π^2^, strongly bent crystals have efficiencies of 45 and 63%, respectively, of the ideal mosaic case, as calculated from equation (7). On the other hand, because of the size of the tiles forming the mosaic structure (Zachariasen, 1945 ▶), deviations from ideal mosaic behaviour are common in real crystals. For example, in a Cu crystal an integrated reflectivity of about 45′′ at 90 keV for the 111 reflection was obtained (Courtois *et al.*, 2005 ▶), corresponding to 58% of the ideal mosaic value as deduced from Fig. 4 ▶. Similarly, a value of *I*_int_/*I*_0_ ≃ 15′′ at 220 keV for the Cu(200) peak was obtained (Loffredo *et al.*, 2005 ▶), corresponding to 39% of the mosaic value. Thus, we are confident that equation (7) may provide a correct comparison between the reflection efficiencies of real mosaic and strongly bent crystals, even if it is not completely reliable for predicting the exact values.

In the range between 80 and 400 keV in the 111 diffraction geometry (lower plot in Fig. 4 ▶), Cu mosaic crystals provide the higher efficiency, nearly twice as large as that of GaAs and Ge (the latter is not reported in the figures due to the superposition of its plot with GaAs). This is not true for the 220 geometry (upper plot in Fig. 4 ▶), in which GaAs and Ge provide a 30% higher intensity than Cu crystals, thus confirming that a higher *Z* does not always correspond to a higher efficiency.

By combining the results of Figs. 3 ▶ and 4 ▶, we can conclude that even relatively light materials, under the condition of optimal length *t*_max_, reach an integrated reflectivity comparable to or better than high-*Z* crystals in the energy range 60–600 keV.

## 4. Preparation of thicker diffracting elements   

In order to increase the performance of the Laue lens, optical elements with a larger dimension *h* are required (see Fig. 1 ▶). Owing to the strain value obtained in the damaged layer by the method of surface treatment (Buffagni *et al.*, 2012 ▶, 2013 ▶), the dimension *h* of the crystal plate cannot exceed a few millimetres for a radius of curvature of several metres.

To overcome such limitations we evaluated the possibility of stacking CDP crystals to obtain thicker crystal elements. This method has already been proposed for neutron monochromators (Frey, 1974 ▶; Alianelli *et al.*, 2004 ▶, and references therein), but the monochromator performance was affected by the difficulty in achieving a good alignment among the crystal elements. Indeed, a slight misalignment may cause different Bragg conditions on the selected diffracting planes, producing multiple diffraction peaks and a broadening of the image in the focal plane of the Laue lens.

Precise control of the value of the curvature, the uniformity of the thickness *h* and the stability of the curvature of the tesserae under stress is essential. In order to allow accurate lattice plane alignment, crystals coming from the same wafer and having exactly the same orientation were selected to be assembled in the stack. Moreover, it is extremely important to obtain a very uniform surface grinding, since an error in the thickness *h* of only 1.5% over the entire area may induce a misalignment of hundreds of arcseconds.

The construction of a Laue lens made of several hundreds of crystals is performed by glueing crystals to a rigid frame. This can be a critical process, because the induced stress can cause crystal deformation or misalignment. The choice of glue must take into account several parameters, such as the polymerization time and the capacity to sustain a large temperature variation for space applications. So-called zero-shrinkage adhesives often have a negative characteristic, at least for space applications, since they change their viscosity according to the level of stress resulting from a load applied externally.

**Figure 5:**
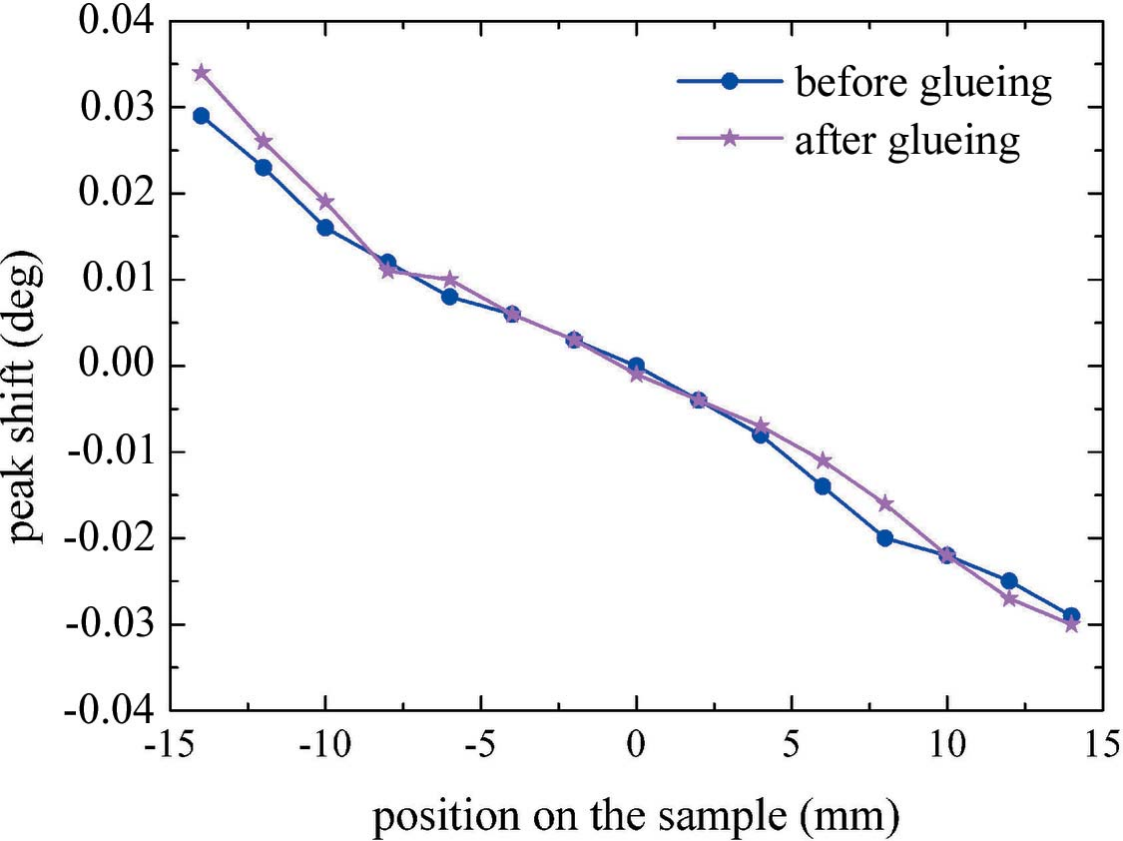
The 004 diffraction peak shifts, as derived from rocking curves measured at different points on the GaAs sample before (blue points) and after (purple stars) glueing. Measurements were performed along the length *t* of the crystal at an energy of 8 keV.

We selected a DEVCON One-Minute epoxy glue with a very fast polymerization time, also used for the Laue project (Virgilli *et al.*, 2013 ▶). To test the effect of the glue on surface-damaged CDP crystals, one tile of GaAs(100) with *R* = 30.2 m and *t* = 28 mm was glued on a glass slide. The glue was placed only at the centre of the sample, to induce a non-uniform stress. High-resolution X-ray diffraction measurements were performed in the Bragg condition at low energy (8 keV). The rocking curve angular shift as a function of the measurement position on the sample gives a direct measure of the sample radius of curvature (Buffagni *et al.*, 2012 ▶). Fig. 5 ▶ shows the 004 rocking curves obtained before and after glueing. The total superposition of measurements with a small deviation at the boundary at *x* = −14 mm demonstrates the negligible effect of the glue on the crystal curvature.

**Figure 6:**
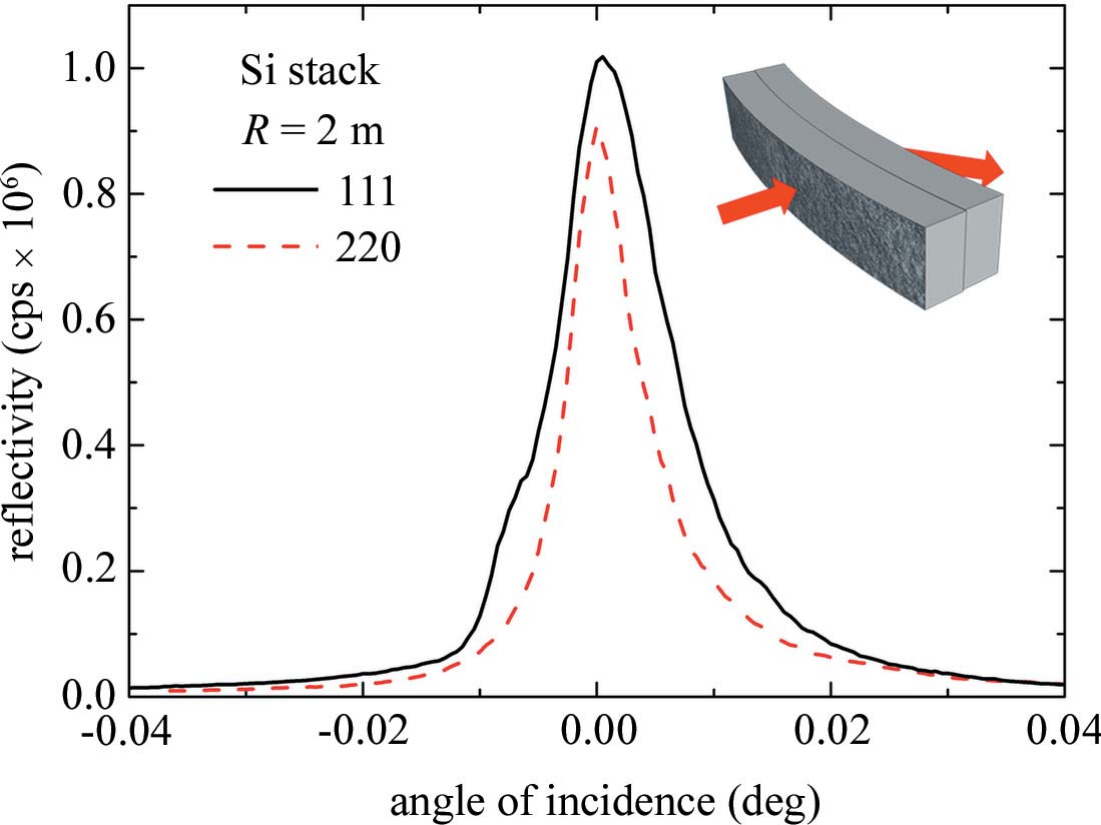
Diffraction profiles of a stack of curved (001) Si crystals measured at 19 keV at the ANKA synchrotron for the 111 and 220 reflections (solid black and dashed red lines, respectively). The inset shows the geometry of the diffraction experiment.

A stack of two Si(001) CDP tiles with dimensions 28 × 8 × 0.5 mm and radius of curvature *R* = 2 m was realized. The high curvature value is necessary to reach the strongly bent regime [equation (5)] even at the low X-ray energies available for the characterization measurements. The alignment of the elements was investigated with an incident beam of 0.5 × 0.5 mm and 19 keV at the PDIFF beamline of the ANKA Synchrotron Radiation Facility (Karlsruhe, Germany) in two different reflections, 111 and 220, and different positions on the tile. Fig. 6 ▶ shows a single and well defined focal spot under X-ray diffraction, confirming that the plates are sufficiently aligned to behave like a single crystal. A similar result was obtained by Neri *et al.* (2013 ▶) with indented Si crystals but with lower curvature values; unfortunately in that case, during the indentation bending technique a large part of the crystal was removed, reducing the hardness and the real thickness of the crystal. It is possible to conclude that the surface-damage bending technique is the most suitable to realize stacked optical elements.

## 5. Conclusions   

Optical elements for X- and γ-ray focusing, made from self-standing curved crystals of Si, GaAs and Ge, were analysed on the basis of the dynamical theory of X-ray diffraction. It was found that CDP crystals with radii of curvature of several tens of metres can be considered as strongly bent crystals for X-ray energies between 60 and 600 keV. In this case, a simplified approach for calculating the integrated reflectivity of the crystals is applied. Under this approximation, the maximum value of the integrated intensity is given by a crystal thickness *t*_max_ = 1/μ, as in the case of ideal mosaic crystals. Then, taking into account the absorption factor, we find that Si, Ge and GaAs crystals with optimized thicknesses show a comparable or superior diffraction efficiency with respect to the heavier materials used previously. This is because the higher structure factor in heavier crystals is compensated for by the larger values of optimized thickness in the Si, Ge and GaAs crystals.

Finally, we have shown that the limitation in the crystal dimension *h* due to the bending technique can be overcome by stacking several curved crystals together. A good alignment to within a few arcseconds between two stacked elements can be achieved by a proper alignment method, permitting crystal elements with a thickness *h* of several millimetres to be obtained.

Thus, it is demonstrated that stacks of Si, Ge and GaAs crystals are optimal optical elements for Laue lenses and pave the way for the realization of new and efficient γ-ray lenses for X-ray astronomy and nuclear medicine.
